# Trapping of Small Molecules within Single or Double Cyclo[18]carbon Rings

**DOI:** 10.3390/molecules28052157

**Published:** 2023-02-25

**Authors:** Natasza Trzęsowska, Rafał Wysokiński, Mariusz Michalczyk, Wiktor Zierkiewicz, Steve Scheiner

**Affiliations:** 1 Faculty of Chemistry, Wrocław University of Science and Technology, Wybrzeże Wyspiańskiego 27, 50-370 Wrocław, Poland; 2 Department of Chemistry and Biochemistry, Utah State University Logan, Logan, UT 84322, USA

**Keywords:** C_18_ ring, dispersion, AIM, NCI, energy decomposition, density shift

## Abstract

The encapsulation of a set of small molecules, H_2_, CO, CO_2_, SO_2_, and SO_3_, by a circular C_18_ ring is investigated by quantum calculations. These ligands lie near the center of the ring but, with the exception of H_2_, are disposed roughly perpendicular to the ring plane. Their binding energies with the C_18_ vary from 1.5 kcal/mol for H_2_ up to 5.7 kcal/mol for SO_2_, and the bonding is dominated by dispersive interactions spread over the entire ring. The binding of these ligands on the outside of the ring is weaker but allows the opportunity for each to bond covalently with the ring. A pair of C_18_ units lie parallel to one another. This pair can bind each of these ligands in the area between them with only small perturbations of the double ring geometry. The binding energies of these ligands to this double ring configuration are amplified by some 50% compared to the single ring systems. The presented data concerning the trapping of small molecules may have larger implications regarding hydrogen storage or air pollution reduction.

## 1. Introduction

Environmental concerns have motivated the continuing development of new green energy sources, reduction of hazardous chemical compounds, and more intelligent uses of natural resources. There is a continuing need to devise new technological approaches in this direction. In the case of the petrochemical industry, replacing petrol fuels with hydrogen involves two main difficulties: storage and transport [1,2,3]. Hydrogen storage methods can be divided into physical methods (compression, liquefaction, or cryocompression) and chemical methods, such as adsorption, conversion into metallic hydrides, complex hydrides and liquid organic carriers [3]. A variety of materials have been examined for these purposes, such as metal organic frameworks, adsorption on many transition metal surfaces, or carbon-based materials. A recent literature report comparing carbon and boron nitride nanotubes indicated that carbon-based nanotubes are more energetically favorable [4].

The work described below focuses on a newly developed carbon allotrope—cyclo[18]carbon (a polyyne-type molecule)—and how noncovalent interactions might enable trapping and storage of small molecule gases within its circular ring structure. Better understanding of these interactions might aid in finding the solutions to current problems such as hydrogen storage and the extraction of harmful gases from the atmosphere. Cyclo[n]carbons have been studied for more than 50 years now [5]. Procedures for the formation of C_18_ from precursors such as C_18_Br_6_ and C_18_(CO)_6_ have been developed and the reaction mechanism is also understood [6,7,8,9]. The C_18_ carbon ring itself consists of 18 carbon atoms bonded with an alternating pattern of single and triple covalent bonds [10]. It is widely recognized that C_n_ rings (where n = 4q + 2 (where q is an integer greater than 1)) containing at least 10 carbon atoms tend to be more stable than their linear chain analogues [5].

Computational studies have shown that this particular kind of polyyne represents the global minimum; the cumulene structure with uniform C–C bond lengths represents the transition state for the bond transposition process from single to triple bonds, and vice versa. At the same time, a bonding structure consisting of carbons connected by double bonds is more aromatic than a polyyne ring due to more effective π orbitals overlapping [11]. This interesting attribute of the cyclo[18]carbon molecule provoked several studies regarding this new carbon allotrope. The electron-accepting potential of the C_18_ ring results from lower C–C bond saturation. Several studies investigated this property of the C_18_ ring along with other cyclocarbons comprising larger or lesser numbers of carbon atoms (from 10 to 60). Research concerning the adsorption of selected gases (CO, NO, NH_3_) has demonstrated that both chemisorption and physisorption are possible [12]. It was also postulated that C_18_ nanoclusters can be used as ultra-fast sensors for detecting CO and NO molecules.

Recent quantum calculations conducted by Hobza et al. proved cyclo[18]carbon’s ability to create dative bonds with piperidine molecules (from one to even four) that attacked from the outside of the C_18_ ring [10]. This interaction was accompanied via significant deformation of the carbon ring. This theme has been extended by the Nandi group [13], which considered the effect of heavy-atom quantum mechanical tunneling on the transformation from a distant van der Waals complex into a more closely bound dative-bond complex at cryogenic temperatures. The energy barrier to this structural reshaping was measured as 2.2 kcal/mol [13] compared to 3.6 kcal/mol in previously cited work [10].

The history of study regarding the interaction between cyclo[n]carbons and piperidine shows that even C_60_ can adsorb one or two piperidine molecules via a N–C dative bond, and the interaction energy of such adducts reaches up to 37 kcal/mol [14]. Other research on this matter involved the examination of the ground and excited states of the C_18_ ring and its complexes with certain electron-donating units such as tetrathiafulvalene, zinc porphyrin, or zinc phthalocyanine [15]. Density functional calculations also explored combinations of cyclo[18]carbon complexes with the noble gases [16], as well as with the XCN (X = H, F, Cl, Br, I) molecules [17]. In the latter paper, the vdW potential map of the C_18_ ring with the halogen probe revealed cylindrical (for F) or dumbbell-shaped (for other halogens) areas for the negative vdW potential, indicating that in this region attraction produced by dispersion forces dominates over exchange repulsion. These spherical forms grew in size from F to I, demonstrating the possibility of diverse objects circling close to C_18_, interacting with this polyyne in various ways. The ability of the Si-substituted cyclo[18]carbon to bind with molecular N_2_ was very recently investigated. The carbon ring had to be bent in order to create the resultant adduct. This reaction can serve as a potential precursor for the further conversion of C_17_Si∙∙∙N_2_ into ammonia [18]. The cyclo[18]carbon species can be viewed as potential materials for hydrogen storage. Therefore, the binding energies of C_18_∙∙∙H_2_/(C_18_)_2_∙∙∙H_2_ adducts might be compared to some other compounds reported earlier in the literature. The growing popularity of graphene motivated its study as a possible hydrogen storage unit. It has been shown that the physisorption energy of molecular hydrogen on flat carbon nanoparticles (graphitic platelets) and polycyclic aromatic hydrocarbons (PAHs) ranged from −0.84 to −1.72 kcal/mol, with the latter value assigned to graphene itself [19]. Other systems potentially useful for hydrogen storage applications were pristine and functionalized GaS sheets, with the binding energies of molecular hydrogen ranging from −1.48 to −8.54 kcal/mol (for pristine GaS it was −1.78 kcal/mol) [20]. Finally, for pristine and decorated GeC monolayers, the binding energies were reported as falling within the scope of −0.23 to −6.81 kcal/mol (the best were for Li and K atom doping) [21].

The purpose of the work described below was to investigate the nature of the interactions between cyclo[18]carbon (single or double) and small gas molecules such as H_2_, CO, CO_2_, SO_2_ and SO_3_, which can provide some fundamental information related to hydrogen storage and air pollution reduction. All the possible complexation routes were considered: both trapping the ligand inside the ring and adsorption from the outside of C_18_. How do these two interaction modes compare? Which gas molecules can be captured within the C_18_ ring? How large can it be? What is the exact nature of these interactions? Is this complexation driven by dispersion or other forces? What is the consequence of adding a second C_18_ ring? Does this addition enhance the binding or change it in a qualitative manner? Insights into the storage process that would occur in a condensed phase are predicated on a thorough understanding of these complexes in the gas phase, where each particular interaction can be studied individually and carefully.

## 2. Results

**Figure 1:**
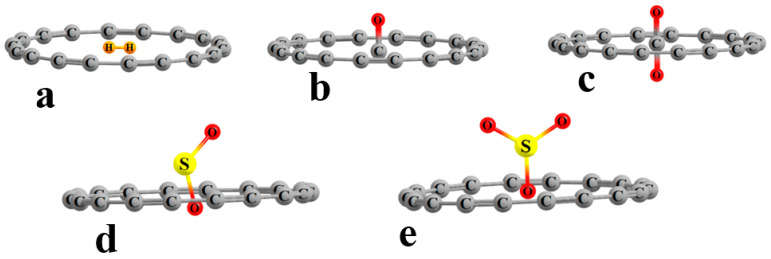
Geometries of complexes pairing C_18_ with (**a**) H_2_, (**b**) CO, (**c**) CO_2_, (**d**) SO_2_, and (**e**) SO_3_.

The structures of the various internal complexes with the ligand situated near the center of the C_18_ ring are exhibited in Figure 1. There are certain differences from one inclusion complex to the next. The H_2_ molecule is small enough that its entirety can fit inside the circle, with both H atoms lying in the C_18_ plane, as seen in Figure 1a. It causes no deformation of the circular nature of the ring, with all the C atoms lying 3.693 Å from the ring center, as indicated in the first row of Table 1. The H_2_ lies slightly off center, with its midpoint displaced 0.062 Å from the ring center. The CO lies perpendicular to the ring plane, with the C within this plane, as may be seen in Figure 1b. Its presence distorts the ring into a slight oval shape, with the maximum and minimum distances from the ring center being 3.695 and 3.689 Å. As indicated in the last column of Table 1, the C atom lies some 0.086 Å from the ring center. The C atom of the CO_2_ also lies in the ring plane, with one O above and the other below, as shown in Figure 1c. The ring becomes more oval in the presence of SO_2_ and SO_3_, with differences between the long and short axes of 0.070 and 0.108 Å, respectively. In both cases, one of the O atoms lies closest to the ring center, with the remainder of the ligand lying above the ring, as shown Figure 1d,e. In the case of SO_2_, the O atom lies significantly below the plane, a full 0.406 Å from its center. Additional calculations for two complexes (C_18_···H_2_ and C_18_···SO_2_) were carried out to consider the freedom of motion of each ligand within its bound environment. In both cases, the rotation barriers of the trapped molecules were estimated around an axis perpendicular to the plane of the ring passing through the center of the H–H bond or the sulfur atom, respectively. The barrier to rotation was found to be 0.58 and 1.21 kcal/mol, respectively. Thus, ligand rotation would require an additional energy equivalent to 38 and 21% of the binding energy for its optimal positioning.

The interaction energies between each ligand and the ring reported in Table 2 indicate a weak to moderate strength. While the E_int_ is less than 2 kcal/mol for H_2_, it rises to 2.8 kcal/mol for CO and exceeds 4 kcal/mol for the other three ligands. The deformation energies E_def_ resulting from the distortions of the two monomer units are quite small, 0.1 kcal/mol or less, as presented in the penultimate column of Table 2, despite the deviations from circularity of some of these rings. Consequently, the binding energy E_b_ listed in Table 2, which equates to the reaction energy of the formation of the inclusion complex from the two isolated monomers, is virtually identical to the E_int_.

One can glean insight into the nature of the interaction via decomposition of the total E_int_ into components with physical meaning. The values of these components in Table 3 suggest these interactions are largely dispersion-controlled, as the E_disp_ accounts for roughly 80% of the total attractive forces, with the electrostatic E_es_ and orbital interaction E_oi_ terms comprising some 10% each.

The AIM protocol is generally a useful tool for pinpointing specific interatomic bonding patterns. The bond paths elucidated for these inclusion complexes are shown in Figure S1 and offer only partial understanding. For example, each of the H atoms of H_2_ would appear to be bonded to a C–C midpoint, and only to those closest to these atoms, with little connection to other C atoms of the ring. The C atoms of CO and CO_2_ each reach out to several ring atoms. The O atoms of SO_2_ and SO_3_ connect to the ring C atoms, despite being displaced above the ring. The weakness of any of these bonds is affirmed by the small densities of each bond critical point, which are in the 0.002–0.004 au range. The other AIM parameters of each complex, such as the ∇^2^ρ and H, are all listed in Table S1, the values of which all suggest a weak noncovalent bond in each case. Moreover, also displayed in Table S1 are the bond critical point quantities for the internal C–C and C≡C bonds of the ring, which are consistent with an alternating weak/strong pattern.

**Figure 2:**
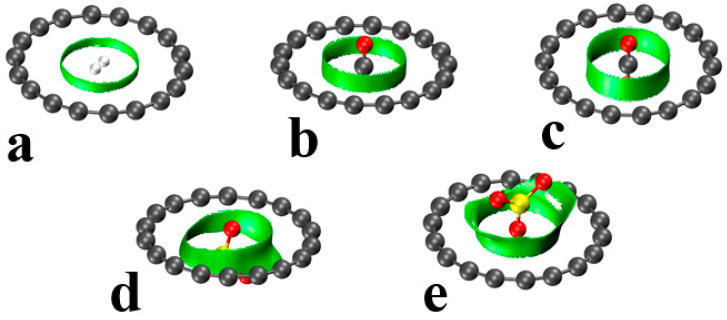
NCI isosurfaces of the C_18_–L (L = (**a**) H_2_, (**b**) CO, (**c**) CO_2_, (**d**) SO_2_, (**e**) SO_3_) dimers (green spheres represent noncovalent interaction regions) at the RDG 0.5 au isovalue.

Noncovalent interaction analysis (NCI), also known as the reduced density gradient method, of these systems offers a perhaps more balanced view of the ligand–ring interactions. The green regions in Figure 2, signaling a weak noncovalent interaction, are generally annular in shape, which suggests a less specific atom–atom bonding than might arise from a survey of the AIM diagrams. So, for example, while AIM proposes that each H atom of H_2_ binds to one specific C ring atom, the NCI pattern in Figure 2a provides a picture in which this bonding is delocalized over the entire ring. There is some asymmetry in the green regions, as in the SO_2_ and SO_3_ cases, which is based on the disposition of the ligand O atoms. Even in those cases, the green area fully encircles the ligand, which is again consistent with the idea that these complexes are dominated by nonspecific dispersive forces.

**Figure 3:**
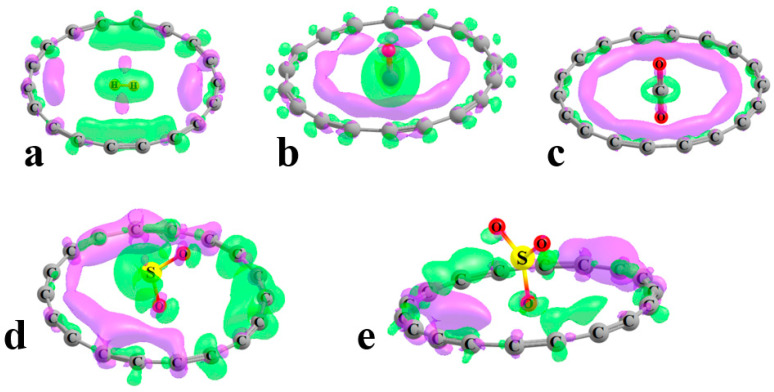
Shifts in electron density caused by complexation. Purple regions correspond to increase, with losses shown in green. The contours in each system are as follows in au: (**a**) H_2_, 0.00005, (**b**) CO, 0.00007, (**c**) CO_2_, 0.00020, (**d**) SO_2_, 0.00030, and (**e**) SO_3_, 0.00030.

Another perspective on the binding arises from examination of how the complexation shifts the electron density around the entire system. The electron density shift patterns illustrated in Figure 3 were derived by subtracting the sum of the unperturbed monomer densities from that of the full complex. Regions where the density has increased are designated by purple, while the green areas indicate loss. A different contour was used for each diagram in order to best display the shift patterns in each, although these contours represent small numbers, between 0.00005 and 0.0030 au, congruent with the lack of largescale shifts.

Figure 3a indicates that there is a shift from the H–H axis of the H_2_ ligand toward the ring atoms that lie generally along this axis. The shift is in the opposite direction, from the ring to H_2_, in the direction perpendicular to the molecular axis. There is a minute shift of overall charge from the ring to H_2_ (computed as the sum of the natural atom charges) of 0.0005 e. This shift is in the opposite direction, from the ligand to the ring for CO, in the amount of 0.0056 e. This shift is evident by the green area encompassing the ligand in Figure 3b and the purple area closer to the C atoms of the ring. This pattern remains quite similar for CO_2_ in Figure 3c, although the larger contour masks a slight transfer to the central ligand of 0.0009 e. The noncylindrical shape of the SO_2_ and SO_3_ ligands introduces the asymmetry of the density shift patterns in Figure 3d,e such that certain regions of the ring accrue additional charge and others lose density. In both cases, there is a small overall shift of charge from the ligand to ring, 0.0085 and 0.0013 e for SO_2_ and SO_3_, respectively, confirmed by the green regions surrounding these ligands.

Concerning the electrostatic portion of the interaction energies, the C_18_ ring is nonpolar for all intents and purposes. The molecular electrostatic potential (MEP) surrounding this system is displayed in Figure S2 on its 0.001 au isodensity surface. The generality of the green area reinforces its nonpolarity. The values of the maximum and minimum on the inside of the ring are respectively +1.8 and −1.4 kcal/mol, as indicated in Table 4. These quantities are of such a small magnitude that they can interact only very weakly with the positive or negative regions of the ligands listed in the table, some of which can be sizable, and are displayed graphically in Figure S2.

In addition to situating themselves in the middle of the C_18_ ring, these ligands can also attach to the outside. The geometries depicted in Figure S3 place the binding C or S atom near the center of a C–C bond of the ring. The C atom of CO, like one of the hydrogen atoms of H_2_, approaches one of the longer C–C single bonds of the ring, whereas it is the shorter triple C≡C bond that attracts the C of CO_2_ or the S of SO_2_ and SO_3_. These outer complexes are significantly weaker than the inner inclusion structures, by a factor of 2 to 6, with their binding energies listed in the first column of Table S2. This weaker binding occurs despite the much larger MEP maximum on the outside of the C_18_ ring, 8.0 vs. 1.8 kcal/mol for the interior maximum.

From this exterior position, it is possible for the ligands to approach the ring more closely so as to form a covalent dative bond with one or more C ring atoms. A certain exception here is the H_2_ molecule, which dissociates before joining the ring, which results in a hydrogenated ring. These products are displayed in Figure S4, along with some of the related energetic quantities. As may be expected, the deformation energies are quite high for both the ring and the erstwhile ligand. The second and third columns of Table S2 summarize the binding energies of the noncovalent and covalent complexes, as well as the activation energy required to convert from the former into the latter. Even though some of the covalently bound complexes are more stable than their noncovalent counterparts, the activation energies are prohibitively high for their formation, in excess of 25 kcal/mol. The exception is SO_3_, where the barrier is only 8 kcal/mol, which would enable the system to be stabilized by some 30 kcal/mol relative to the noncovalent complex.

### Double Ring

**Figure 4:**
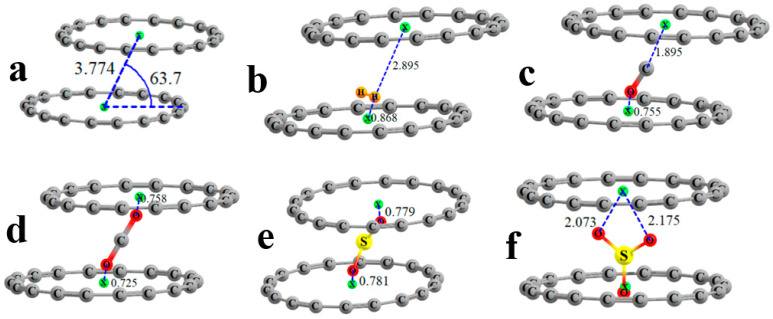
Geometries of double ring systems with ligands: (**a**) none, (**b**) H_2_, (**c**) CO, (**d**) CO_2_, (**e**) SO_2_, and (**f**) SO_3_. Green spheres indicate center of each ring, distances in Å.

The flat shape of the C_18_ ring begs the question of whether two such rings might dimerize. An optimization of such a dimer leads to the structure pictured in Figure 4a, which can be categorized as a slipped parallel geometry, reminiscent of the favored geometry of the benzene dimer. The centers of the two rings, indicated by the small green balls, lie some 3.774 Å from one another, and the center-to-center axis makes an angle of 63.7° with the plane of each ring, which places the planes of the two units 3.38 Å apart, as indicated by the d quantity in the last column of Table 5. The interaction energy between the two rings in this dimer amounts to −8.96 kcal/mol.

The geometries of the complexes in which each ligand is inserted into the C_18_ dimer systems are represented in Figure 4b–f. In the cases of H_2_, CO, and SO_3_, the ligand is asymmetrically positioned, being much closer to one ring than the other. For example, one H atom of H_2_ lies 0.9 Å from the center of the lower ring but nearly 3 Å from the upper. By contrast, the terminal O atoms of CO_2_ are each some 0.7 Å from the upper and lower rings, respectively, with similar distances noted in SO_2_. In most cases, the ligand adopts a very different position with the triad than with the single ring. H_2_ is pulled out of the plane of the lower ring, as are CO and CO_2_, which are also tilted away from the vertical. SO_2_ and especially SO_3_ seem to be less affected by the presence of the upper ring.

The energetics of adding each ligand to the double ring compiled in Table 5 correspond to the interaction energy between the ligand and the pair of rings, all in the geometry they adopt within the triad. These quantities span the range between 2.2 kcal/mol for H_2_ and 7.4 kcal/mol for SO_3_. These triad interaction energies represent a roughly 50% increase when compared to the single-ring complexes in Table 2. The deformation energies in Table 5 are somewhat larger than those in Table 2, signaling that the inclusion of the ligand has a larger effect on the double ring geometry than it does on the single ring. A good part of this distortion has to do with the pushing of the two rings apart by the ligand. The distance between the ring planes reported in the last column of Table 5 can be compared with the 3.38 Å in the unliganded ring dimer, which is closely related to the deformation energy of the system.

An energy decomposition of each of these triads, as presented in Table 6, shows that like the single ring systems, so too are the larger triads dominated by dispersion, which accounts for 70–80% of the total attractive energy. The AIM diagrams of these complexes are exhibited in Figure S5 and show certain similarities to those of the single ring dyads. Bond path densities connecting ligand atoms to those of the ring lie in the range between 0.003 and 0.005 au, and again one sees an alternating pattern of ring C–C and C≡C bonds with densities of 0.33 and 0.41 au, respectively. The NCI plots in Figure S6 are again characterized by delocalized bonding involving the entire ring, rather than any one specific C atom.

## 3. Discussion

There has been a bit of controversy regarding the bonding nature of cyclo[18]carbon, in that some calculations confirmed a polyyne structure [10,11] with alternating short and long C–C bonds, as found experimentally [22], while others suggested a cumulene geometry with uniform bond lengths [11,23]. While our own ωB97XD/Def2TZVPP computations fell in line with the experimental polyyne idea, which has been further quantified as being 10 kcal/mol more favorable than the cumulene [11], our pilot calculations with the PBE0 functional falsely suggested the alternate. It would seem that the bonding pattern within this system is rather sensitive to the particular level of calculation.

The electron-acceptor properties of C_18_ were studied in the work of Hobza et al. in C_18_ complexes with piperidine [10], which also bolsters confidence in the level of theory applied here. After applying this same ωB97XD/Def2TZVPP level [10] in their work on piperidine complexes with different carbon ring systems, these authors also performed single-point energy calculations with the coupled cluster single and double CCSD/cc-pVTZ level and concluded that “The DFT values are in reasonable agreement with the more accurate CCSD ones”. In addition to the vdW complex for which the binding energy was −3.6 kcal/mol, a complex stabilized by a coordination bond (dative bond) was also examined, for which the E_b_ was −16.2 kcal/mol. Our own calculations were consistent in that it was found that a noncovalently bound ligand on the outside of the C_18_ ring could approach more closely and engage in short covalent bonds with two C atoms. The energetics of this process presented in Table S2 show that this process can be an exothermic one, for example, as much as −33.6 kcal/mol in the case of SO_3_. However, this conversion from a noncovalent to a covalent complex must overcome an energy barrier. This barrier ranges from 8.1 kcal/mol for SO_3_ up to nearly 50 kcal/mol for CO_2_ and even 135 kcal/mol for H_2_, which, as mentioned before, dissociates before joining the ring. This sort of high barrier contrasts with the previously published barrier of only 3.6 kcal/mol for piperidine. This earlier set of computations did not consider complexes with the piperidine located within the ring, probably due to the fact that piperidine is too large to fit inside the C_18_ ring. It was found here that a similar restriction already applies even to benzene. When placed initially in the center of the ring, benzene was quickly expelled in an exothermic process.

Nandi et al. confirmed that the connection of piperidine to the C_18_ ring requires overcoming an energy barrier, this time estimated at 2.2 kcal/mol [13]. Their work also showed that among the dative bond complexes of various polyynes with piperidine, the one with a 14-member carbon ring was the most stable, while the most stable vdW complex was that with C_16_. In a paper by Vadalkar et al., the dative bond complexes between CO and the C_18_ ring were discussed in detail [12]. The results obtained at the ωB97XD/6-311++G(d, p) level for the C_18_∙∙∙CO adduct were essentially the same as those obtained by the current work (E_b_ = −7.1 kcal/mol).

Mazumder et al. studied the ability of a double C_18_ ring to encapsulate diatomic noble gas molecules at the M06-2X/def2-TZVP level [16]. The binding energies ranged from −2.4 to −6.4 kcal/mol, indicating the most stable adduct for Kr_2_, and this quantity increased in parallel with the size of the noble gas. The energetics of these dimers are similar to the small molecules examined in the current work, with the E_b_ being between −2.2 and −8.7 kcal/mol. The nature of the ring∙∙∙ligand complexes revealed by an SAPT2 decomposition scheme was also consistent with our findings in that the dispersion forces greatly outweighed the electrostatics and induction, with the latter two combining for less than 30% of the total attractive forces.

Different binding modes connecting C_18_ to HCN were examined by Vadalkar et al. [24]. The interaction energy between HCN and the bare ring was roughly −5 kcal/mol and the N∙∙∙C binding distance equal to 3.01 Å. This adsorption energy was found to rise to nearly −14 kcal/mol when these nanoclusters were doped with Al, Si, and P atoms. Similar clusters were examined by Zhao [17], extending the set of ligands to XCN molecules. Both inside and outside binding modes were confirmed for ClCN, BrCN and ICN, while HCN and FCN were limited to only inside the carbon ring. The inner complexes were more stable than the outside ones, much like in our data. While the interaction energies did not surpass −2.4 kcal/mol for the latter, these quantities ranged from −5 to −8 kcal/mol. SAPT analysis confirmed that these dimers are primarily dominated by dispersion. Green rims indicative of the noncovalent interactions typical of vdW complexes, comparable to those seen in Figure 2, were detected via NCI analysis.

As mentioned in the Introduction, the physisorption energy of molecular hydrogen on flat carbon nanoparticles ranged from −0.84 to −1.72 kcal/mol [19]. Our computed H_2_ binding energy by a single ring of −1.51 kcal/mol fits into this range, while the −2.22 kcal/mol for the double ring is superior.

The binding energies can be connected with complexation constants using the approximate expression that K = exp(−E_b_/RT). Since all the E_b_ are negative, these association constants will all exceed unity. Assuming a temperature of 298 K, these constants with the single ring will vary from a minimum of 13 for H_2_ up to 1.6 × 10^4^ for SO_2_. These values increase for the double-ring complexes, with a range of between 42 and 2.3 × 10^6^. K rises markedly as the temperature is diminished. Taking 100 K as an example, this quantity varies between 2.0 × 10^3^ and 3.2 × 10^12^ for the single-ring complexes. These large constants, in tandem with the lack of any energy barrier to their formation, suggests the spontaneity of the association reactions.

It might be noted from Table S3 that the zero-point vibrational energy corrections to the interaction energies are small, in the order of 1 kcal/mol, and so have little effect on the conclusions outlined above. Another issue relates to the low-frequency vibrational modes of some of these systems. Taking the single C_18_ ring as an example, the mode with the smallest frequency of 59 cm^−1^ corresponds to an in-plane deformation that takes the circular shape toward an oval. The next lowest frequency of 76 cm^−1^ represents an out-of-plane vibration. With regard to the double ring, there is a very low-frequency mode of 5 cm^−1^ that can be described as a synchronized swinging of two rings and a second of 5 cm^−1^ involving opposite twists.

## 4. Methods

Calculations were performed at the ωB97XD/Def2TZVPP level of theory [25,26]. Harmonic frequency analysis confirmed that the optimized geometries represent true minima on the potential energy surfaces, without any imaginary frequencies, except of course for those scenarios where a transition state was sought. Numerous starting positions (for example, 5 and 7 initial starting geometries for the C_18_∙∙·H_2_ and C_18_∙∙·SO_2_ dimers, respectively) were considered in the optimizations of each small molecule with a single ring to be sure all the minima were discovered. As per our interest in the trapping of these molecules by multiple rings, optimization starting points for the double rings placed the small molecule in the area between them. The binding energies were calculated by subtracting the sum of the electron energies of the fully isolated monomers from the energy of the complex. The interaction energies included in a parallel formulation the energy of monomers within the geometry of the complex. The difference between these two quantities represents the deformation (or preparation) energy. The interaction energy (E_int_) of each complex was corrected for the basis set superposition error (BSSE) via the counterpoise procedure [27]. Calculations were performed using Gaussian code 16, Rev. C.01 [28]. The extrema values of the molecular electrostatic potential (MEP) on an isodensity surface of 0.001 au were evaluated using the MultiWFN program [29,30]. The MEP maps were visualized using the VMD program [31]. MultiWFN software was also utilized for the NCI analyses [30,32,33], which illustrated and quantified the noncovalent interaction zones. Bader’s AIM methodology encoded within the AIMAll suite of programs was implemented to elucidate the bond paths and to determine their topological properties [34,35,36]. Decomposition of the interaction energies was carried out at the BLYP-D3/ZORA/TZ2P level of theory using the ADF–EDA procedure according to the Morokuma–Ziegler scheme embedded in ADF software [37,38,39].

## 5. Conclusions

The circular C_18_ ring is capable of enclosing a range of small molecules with only very minor perturbations to its internal geometry. These ligands sit more or less at the center of the ring. The binding energies range from 1.5 kcal/mol for the small nonpolar H_2_ up to more than 5 kcal/mol for the larger and more polar SO_2_ and SO_3_. The interactions are dominated by dispersive forces involving the entire ring. Although more weakly bound, the ligands can also attach to the outside of the ring. From this point, they can form covalent bonds to the ring, although such a process must overcome a significant energy barrier. A pair of C_18_ rings situate themselves in a slipped parallel arrangement. This double ring can enclose the same set of ligands, with interaction energies roughly 50% higher than in the case of a single ring. Some of these small molecule lie much closer to one ring, while others are located roughly equally spaced between the two rings.

## Figures and Tables

**Table 1:** Distance (Å) from center of C_18_ ring to C atoms of ring or ligand atom.

L	Min.	Max.	Average	Difference (Max.–Min.)	Distance between Ligand and Ring Center
None	3.693	3.693	3.693	0	-
H_2_	3.689	3.695	3.692	0.006	0.062 ^a^
CO	3.691	3.694	3.693	0.003	0.086 ^b^
CO_2_	3.689	3.695	3.692	0.006	0.012 ^b^
SO_2_	3.655	3.725	3.690	0.070	0.406 ^c^
SO_3_	3.640	3.748	3.694	0.108	0.060 ^c^

^a^ Midpoint of H_2_, ^b^ C atom, ^c^ O atom.

**Table 2:** Interaction, binding, and deformation energies (kcal/mol) of C_18_···L inclusion complexes calculated at the ωB97XD/Def2TZVPP level of theory.

L	E_int_	E_def_	E_b_
H_2_	−1.48 (−1.51) ^a^	0.00	−1.51
CO	−2.84 (−2.96)	0.00	−2.96
CO_2_	−4.12 (−4.36)	0.00	−4.36
SO_2_	−4.50 (−5.79)	0.06	−5.73
SO_3_	−4.81 (−5.35)	0.11	−5.24

^a^ Without BSSE correction.

**Table 3:** EDA/BLYP-D3/ZORA/TZ2P decomposition of the interaction energy of C_18_ complexes into Pauli repulsion (E_Pauli_), electrostatic (E_elec_), orbital interaction (E_oi_) and dispersion (E_disp_) components. All energies in kcal/mol.

L	E_Pauli_	E_es_	% ^a^	E_oi_	%	E_disp_	%	Total
H_2_	2.07	−0.42	11	−0.38	10	−3.20	79	−1.93
CO	4.67	−0.99	10	−0.97	10	−7.51	80	−4.79
CO_2_	4.27	−0.80	7	−0.87	8	−9.11	85	−6.52
SO_2_	8.13	−2.25	13	−2.01	12	−13.01	75	−9.14
SO_3_	7.40	−2.36	14	−1.30	8	−13.63	78	−9.89

^a^ Percentage contribution to total attractive interactions (E_elec_ + E_oi_ + E_disp_).

**Table 4:** Extrema of MEP (kcal/mol) calculated at ωB97XD/Def2TZVPP level of theory.

Molecule	V_s,max_	V_s,min_
C_18_	8.0 ^a^, 1.8 ^b^	−1.4
(C_18_)_2_	8.0 ^a^, 2.8 ^b^	−2.3
H_2_	10.5	−2.7
CO	11.4	−6.4 ^c^, −13.3 ^d^
CO_2_	27.0	−12.6
SO_2_	34.3	−19.9
SO_3_	55.2	−10.9

^a^ Outside of ring. ^b^ Inside of ring. ^c^ On O atom. ^d^ On C atom.

**Table 5:** Interaction, binding, and deformation energies (kcal/mol) of C_18_∙∙·L∙∙·C_18_ inclusion complexes calculated at the ωB97XD/Def2TZVPP level of theory. In addition, also listed as d (Å) is the distance between ring planes.

L	E_int_	E_def_	E_b_	d
H_2_	−2.18 (−2.23) ^a^	0.01	−2.22	3.38
CO	−4.30 (−4.53)	0.17	−4.36	3.44
CO_2_	−6.27 (−6.74)	0.09	−6.65	3.41
SO_2_	−7.48 (−9.10)	0.41	−8.69	3.48
SO_3_	−7.37 (−8.28)	1.04	−7.24	3.64

^a^ Without BSSE correction.

**Table 6:** EDA/BLYP-D3/ZORA/TZ2P decomposition of the interaction energy of complexes into Pauli repulsion (E_Pauli_), electrostatic (E_elec_), orbital interaction (E_oi_) and dispersion (E_disp_) components. All energies in kcal/mol.

	E_Pauli_	E_elec_	% ^a^	E_oi_	%	E_disp_	%	E_int_
(C_18_)_2_∙∙∙H_2_	3.18	−0.75	13	−0.52	9	−4.51	78	−2.61
(C_18_)_2_∙∙∙CO	6.88	−1.71	12	−1.34	10	−10.72	78	−6.89
(C_18_)_2_∙∙∙CO_2_	7.69	−2.11	12	−1.36	8	−13.80	80	−9.59
(C_18_)_2_∙∙∙SO_2_	15.05	−4.61	16	−4.20	14	−20.40	70	−14.16
(C_18_)_2_∙∙∙SO_3_	16.53	−5.80	18	−2.72	8	−24.41	74	−16.40

^a^ Percentage contribution to total attractive interactions (E_elec_ + E_oi_ + E_disp_).

## Data Availability

The data presented in this study are available in supplementary material.

## Supplementary Materials

The following supporting information can be downloaded at: https://www.mdpi.com/article/10.3390/molecules28052157/s1, Figure S1: QTAIM molecular diagrams of C_18_···L (L = H_2_, CO, CO_2_, SO_2_, SO_3_) dimers. Green dots represent bond critical points. Numbers refers to electron densities at BCPs (in au); Figure S2: MEP of the monomers (C_18_, H_2_, CO, CO_2_, SO_2_, SO_3_) on the 0.001 au isodensity surface. Color scale is −0.10 au (blue) to 0.10 au (red); Figure S3: Geometries of complexes with ligands attached to the outside of the C_18_ ring. Distances in Å; Figure S4: Covalent cyclo[18]carbon dimers with ligands along with their binding and deformation energies given in kcal/mol. Distances in Å.; Figure S5: AIM molecular diagrams of (C_18_)_2_···L complexes. Green dots represent bond critical points. Numbers refers to electron densities at BCPs (in au); Figure S6: NCI isosurfaces of the (C_18_)_2_-L (complexes at the RDG 0.5 au isovalue.; Table S1: AIM descriptors of the calculated complexes. Bond critical point (BCP) properties: electron density *ρ*, Laplacian of electron density ∇^2^*ρ* and total electron energy H and potential electron density energy V as well as kinetic electron density energy G, were obtained at the ωB97XD/Def2TZVPP level. Data in atomic units; Table S2: Binding energies (kcal/mol) of ligands attached to the outside of the C_18_ ring, and barrier to convert from noncovalent to covalent; Table S3: Zero-point vibrational energies, and fully corrected interaction energies (kcal/mol); Table S4: Coordinates of studied systems.
