# Supplementary material for: Trapping of Small Molecules within Single or Double Cyclo[18]carbon Rings
Source: Molecules. 2023 Feb 25;28(5):2157. doi: 10.3390/molecules28052157 (PMC10004474; doi:10.3390/molecules28052157)
Supplement: Supplementary file 1 [file molecules-28-02157-s001.zip › molecules-2206709-supplementary.pdf]

SUPPLEMENTARY INFORMATION

Trapping of Small Molecules within Single or Double Cyclo[18]carbon Rings

Natasza Trzęsowska [1], Rafał Wysokiński [1], Mariusz Michalczyk [1,*], Wiktor Zierkiewicz [1,*] and Steve Scheiner [2]

1 Faculty of Chemistry, Wrocław University of Science and Technology, Wybrzeże Wyspiańskiego 27, 50-370 Wrocław, Poland; natasza.trzesowska@pwr.edu.pl (N.T.); rafal.wysokinski@pwr.edu.pl (R.W.)

2 Department of Chemistry and Biochemistry, Utah State University Logan, Logan, UT 84322, USA; steve.scheiner@usu.edu

* Correspondence: mariusz.michalczyk@pwr.edu.pl (M.M.); wiktor.zierkiewicz@pwr.edu.pl (W.Z.)

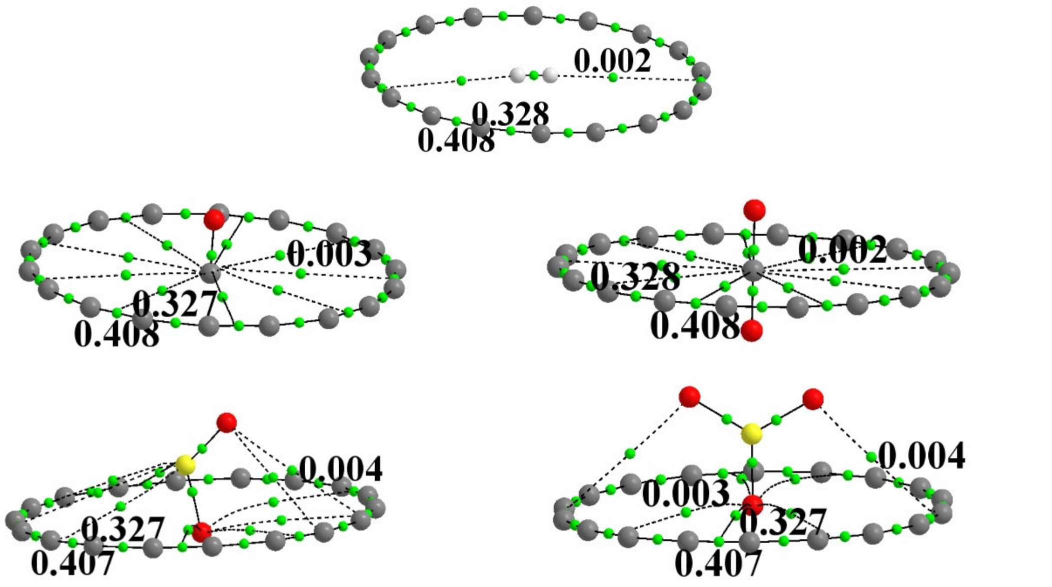

Figure S1. QTAIM molecular diagrams of $C_{18}\cdots L$ (L = H_2, CO, CO_2, SO_2, SO_3) dimers. Green dots represent bond critical points. Numbers refers to electron densities at BCPs (in au).

Table S1. AIM descriptors of the calculated complexes. Bond critical point (BCP) properties: electron density ρ, Laplacian of electron density $^2\rho$ and total electron energy H and potential electron density energy V as well as kinetic electron density energy G, were obtained at the ωB97XD/Def2TZVPP level. Data in atomic units.

	**I~~i~~nteraction**	ρ	$\nabla^2\rho$	**H**	**V**	**G**
C_{18}-H_2	C_r–C_r	0.328	−0.977	−0.371	−0.498	0.127
	C_r≡C_r	0.408	−1.210	−0.572	−0.842	0.270
	H···C_r	0.002	0.009	0.001	−0.001	0.002
C_{18}-CO	C_r–C_r	0.327	−0.977	−0.371	−0.498	0.127
	C_r≡C_r	0.408	−1.208	−0.572	−0.841	0.270
	C_r···C	0.003	0.011	0.001	−0.001	0.002
	C=O	0.509	0.903	−0.947	−2.119	1.172
C_{18}-CO_2	C_r–C_r	0.408	−1.208	−0.572	−0.842	0.270
	C_r≡C_r	0.328	−0.977	−0.371	−0.498	0.127
	C_r···C	0.002	0.010	0.001	−0.001	0.002
	C=O	0.470	0.293	−0.874	−1.821	0.947
C_{18}-SO_2	C_r–C_r	0.327	−0.976	−0.371	−0.498	0.127
	C_r≡C_r	0.408	−1.207	−0.572	−0.841	0.270
	S=O	0.308	1.240	−0.349	−1.008	0.659
	C_r···O	0.004	0.015	0.001	−0.002	0.003
	C_r···S	0.004	0.015	0.001	−0.002	0.003
C_{18}-SO_3	C_r–C_r	0.328	−0.980	−0.372	−0.499	0.127
	C_r≡C_r	0.409	−1.217	−0.574	−0.843	0.269
	S=O	0.319	1.154	−0.378	−1.044	0.666
	C_r···O	0.003	0.012	0.001	−0.001	0.002
	C_r···O′	0.004	0.018	0.001	−0.002	0.003

C_{18}

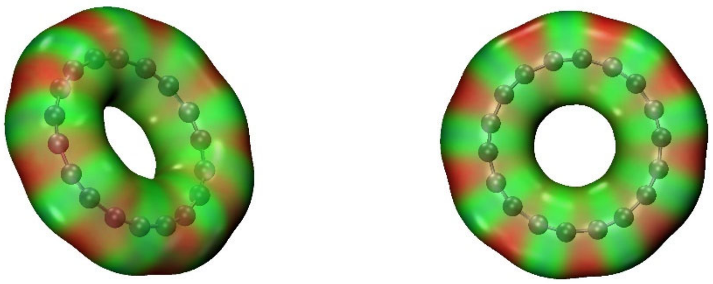

H_2

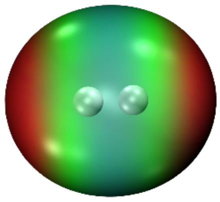

CO

CO_2

SO_2

SO_3

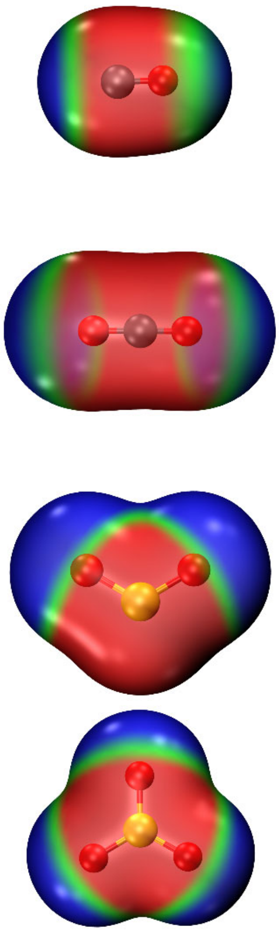

Figure S2. MEP of the monomers (C_{18}, H_2, CO, CO_2, SO_2, SO_3) on the 0.001 au isodensity surface. Color scale is −0.10 au (blue) to 0.10 au (red).

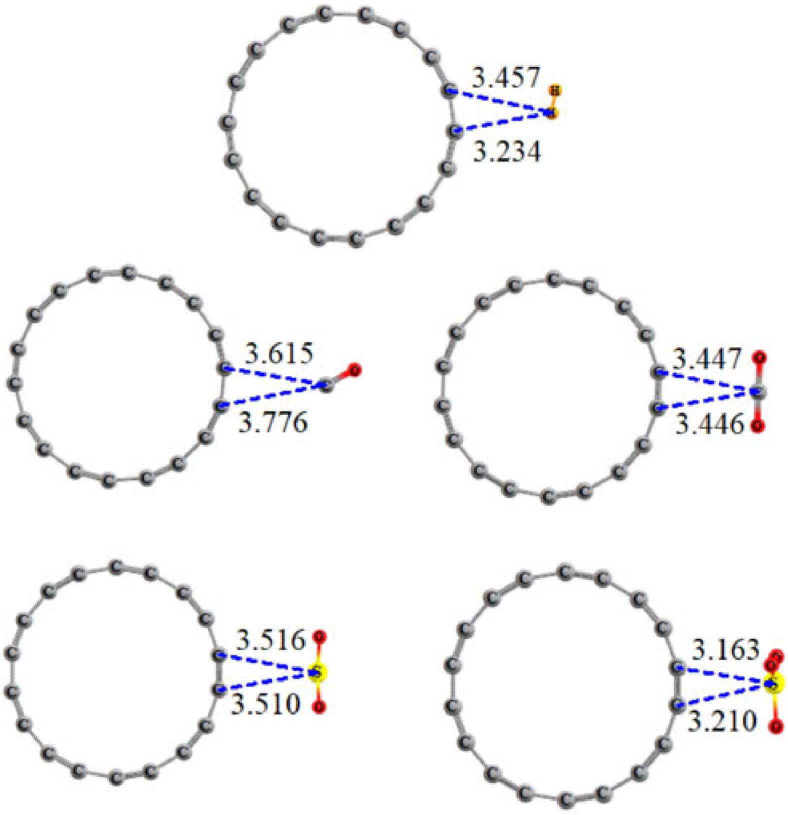

Figure S3. Geometries of complexes with ligands attached to the outside of the C_{18} ring. Distances in Å.

Table S2. Binding energies (kcal/mol) of ligands attached to the outside of the C_{18} ring, and barrier to convert from noncovalent to covalent.

	noncovalent	covalent	transition state
L	E_b	E_b	$E^{\dagger}$
H_2	−0.31	−64.46	135.46
CO	−0.50	−7.13	25.81
CO_2	−1.15	1.72	47.47
SO_2	−1.70	−15.14	31.48
SO_3	−2.88	−33.62	8.09

Covalent complexes	E_b	$E_{def}(C_{18})$	E_{def}(ligand)
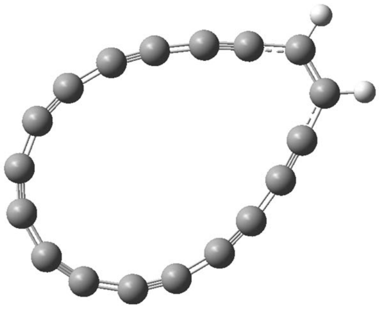	−64.46	49.92	139.71
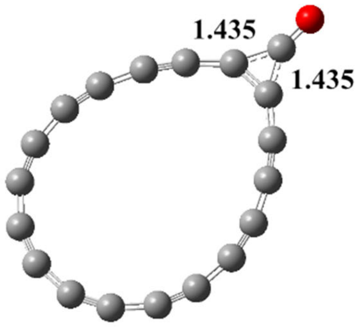	−7.13	30.14	5.67
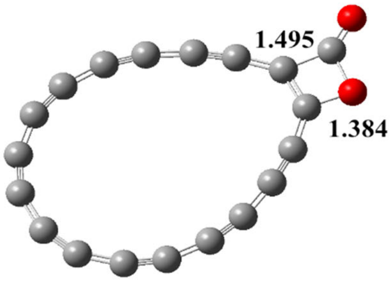	1.72	33.87	90.99
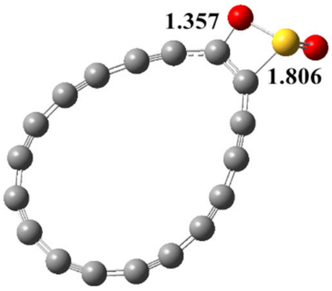	−15.14	36.46	46.17
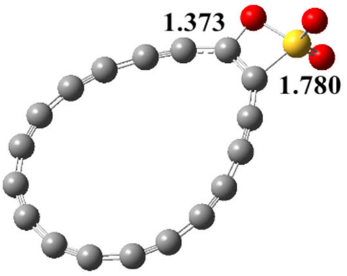	−33.62	34.96	58.81

Figure S4. Covalent cyclo[18]carbon dimers with ligands along with their binding and deformation energies given in kcal/mol. Distances in Å.

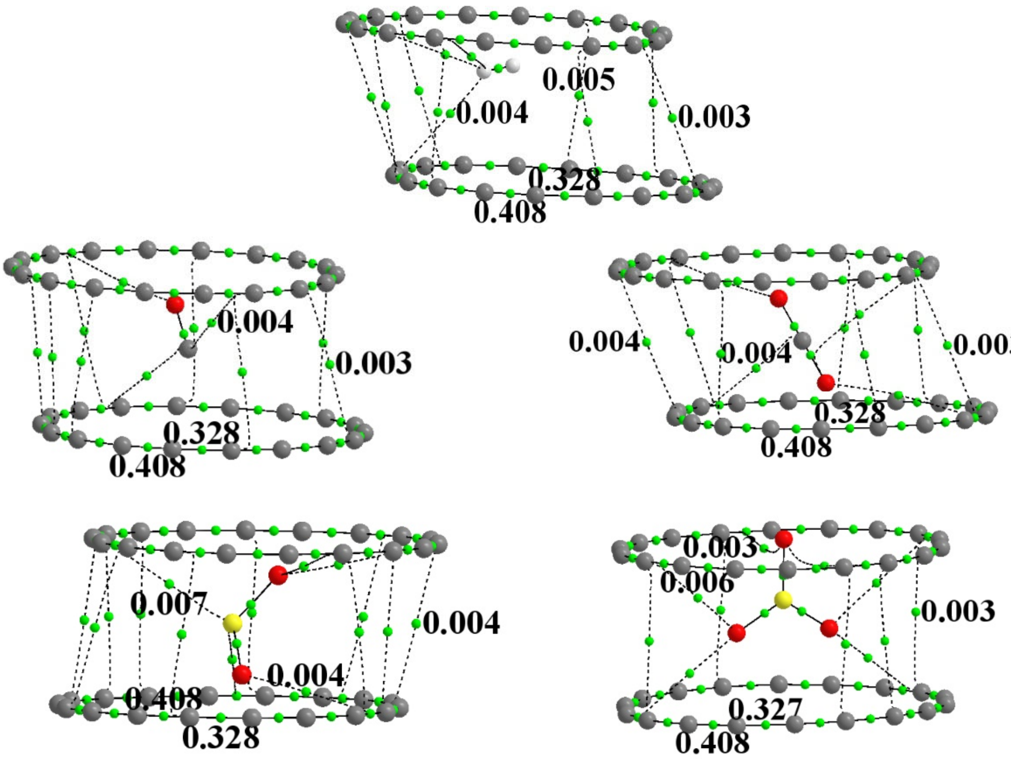

Figure S5. AIM molecular diagrams of $(C_{18})_2\cdots L$ complexes. Green dots represent bond critical points. Numbers refers to electron densities at BCPs (in au).

$(C_{18})_2$-H_2

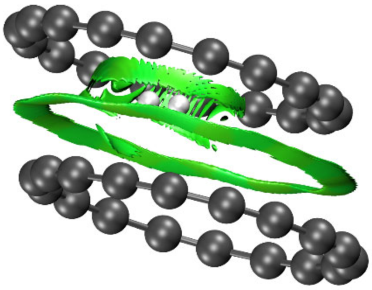

$(C_{18})_2$-CO

$(C_{18})_2$-CO_2

$(C_{18})_2$-SO_2

$(C_{18})_2$-SO_3

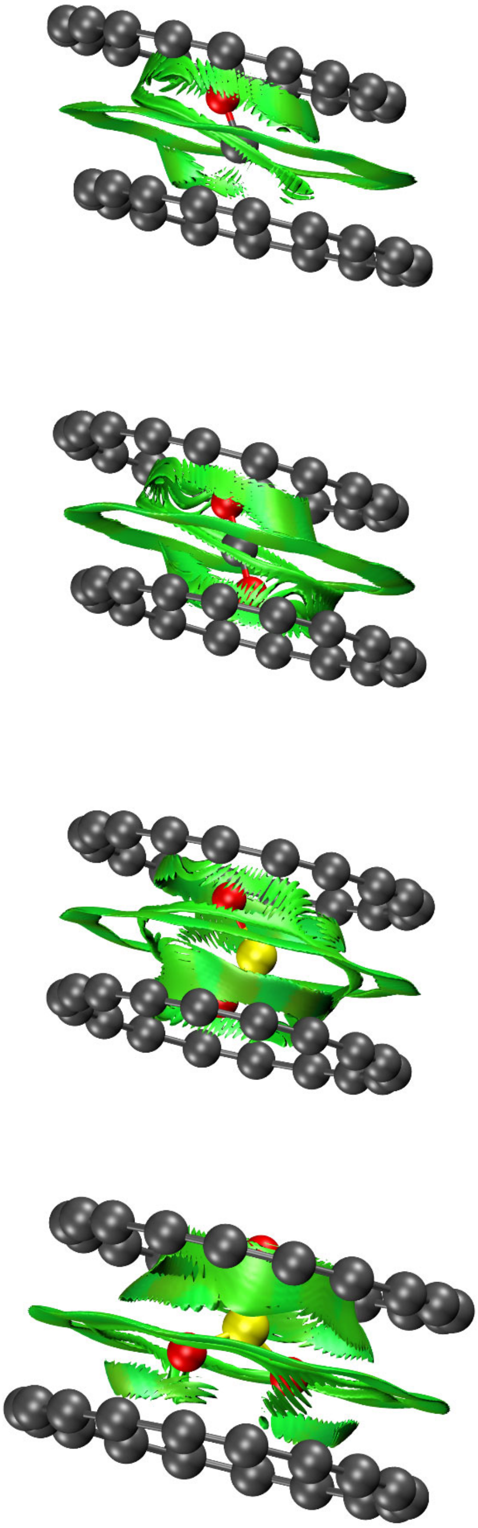

Figure S6. NCI isosurfaces of the $(C_{18})_2$-L (complexes at the RDG 0.5 au isovalue.

Table S3. Zero-point vibrational energies, and fully corrected interaction energies (kcal/mol).

**Dimer (one ring)**				Trimer (two rings)			
**L**	**E_{int}**	**ZPVE**	**$E_{int(ZPVE)}$**	L	E_{int}	ZPVE	$E_{int(ZPVE)}$
H_2	−1.48	0.81	−0.67	H_2	−2.18	0.78	−1.40
CO	−2.84	0.35	−2.49	CO	−4.3	0.39	−3.91
CO_2	−4.12	1.28	−2.84	CO_2	−6.27	1.34	−4.93
SO_2	−4.50	0.33	−4.17	SO_2	−7.48	0.15	−7.33
SO_3	−4.81	0.39	−4.42	SO_3	−7.37	0.75	−6.62

Table S4. Coordinates of studied systems:.

a) Internal coordinates of ωB97XD/Def2TZVPP optimized C_{18} and C_{18} dimer.

C_{18}	6	−4.815041	0.348934	0.057063
	6	−3.621235	0.125045	0.176823
	6	−2.289785	0.289707	0.089022
	6	−1.211979	0.794824	−0.180808
	6	−5.955027	0.977577	−0.278868
	6	−6.706347	1.825988	−0.732042
	6	−0.311996	1.675717	−0.651602
	6	−7.121451	2.953920	−1.334596
	6	0.145485	2.673645	−1.184954
	6	−7.078405	4.029779	−1.909312
	6	0.192767	3.858708	−1.818200
	6	−0.184212	4.882599	−2.365160
	6	−6.574556	5.129257	−2.496592
	6	−5.757403	5.928932	−2.923725
	6	−1.011809	5.817257	−2.864226
	6	−4.570306	6.485702	−3.221032
	6	−2.046549	6.388199	−3.169020
	6	−3.361587	6.634977	−3.300738
$(C_{18})_2$	6	−0.505163	−3.381409	−0.763547
	6	0.287898	−4.303769	−0.864930
	6	−0.988881	−0.913150	−0.536876
	6	−0.977523	−2.129024	−0.642755
	6	4.961991	1.412912	−0.753392
	6	3.901034	2.003395	−0.629485
	6	2.579603	2.214911	−0.506625
	6	1.383362	1.984175	−0.430875
	6	0.229375	1.295574	−0.409023
	6	−0.543461	0.351522	−0.446521
	6	1.458974	−4.956240	−0.960634
	6	2.662232	−5.150100	−1.027820
	6	3.981440	−4.898334	−1.081457
	6	5.029594	−4.273439	−1.101551
	6	5.879258	−3.232012	−1.089029
	6	6.281947	−2.080374	−1.050668
	6	5.835411	0.399384	−0.880312
	6	6.266689	−0.738659	−0.974294
	6	−0.374102	−3.120819	2.707868
	6	0.792719	−3.772730	2.567705
	6	−1.638474	−0.946532	2.931806
	6	−1.167427	−2.200786	2.825921
	6	3.244149	3.182581	2.910741
	6	1.919845	3.397871	2.990146
	6	0.721622	3.170139	3.035157
	6	−0.433812	2.483695	3.056131
	6	−1.207091	1.539479	3.047194
	6	−1.652161	0.272133	3.000465
	6	1.992760	−3.965048	2.454877
	6	3.310327	−3.712905	2.373736
	6	4.359421	−3.089201	2.354294
	6	5.212356	−2.051400	2.395082

	6	5.619366	−0.903708	2.478744
	6	5.607209	0.434843	2.598019
	6	4.306202	2.587317	2.823024
	6	5.180494	1.572898	2.709821
b) Internal coordinates of ωB97XD/Def2TZVPP optimized complexes with single ring (ligand within).				
C_{18}-H_2	6	−3.979871	−0.102389	0.001924
	6	−2.644900	−0.231672	−0.089757
	6	−1.481652	0.038380	−0.341551
	6	−0.407847	0.723963	−0.770293
	6	−5.094814	0.385258	−0.092296
	6	−6.068872	1.266304	−0.379182
	6	0.259007	1.626146	−1.250614
	6	−6.614557	2.283636	−0.775269
	6	0.565864	2.806394	−1.816039
	6	−6.772324	3.507673	−1.308041
	6	0.424168	3.917886	−2.299784
	6	−0.178018	5.037924	−2.735845
	6	−6.492647	4.579006	−1.821268
	6	−5.757769	5.572363	−2.350467
	6	−1.061699	5.838543	−2.996422
	6	−4.783465	6.195706	−2.739835
	6	−2.289522	6.375817	−3.100445
	6	−3.501634	6.491416	−3.016447
	1	−3.281962	2.792152	−1.362856
	1	−2.915914	3.350595	−1.690555
C_{18}-CO	6	−4.882160	0.109678	−0.136353
	6	−3.723012	−0.263029	−0.051547
	6	−2.382559	−0.254540	−0.152898
	6	−1.254358	0.133179	−0.410809
	6	−5.940797	0.890183	−0.414206
	6	−6.587844	1.848954	−0.803746
	6	−0.258962	0.926413	−0.843272
	6	−6.868086	3.053217	−1.331385
	6	0.311369	1.892612	−1.323706
	6	−6.700069	4.148575	−1.842885
	6	0.496664	3.099769	−1.885526
	6	0.241886	4.193376	−2.363794
	6	−6.071586	5.212513	−2.372226
	6	−5.168199	5.933016	−2.765183
	6	−0.470746	5.249535	−2.792675
	6	−3.925386	6.359879	−3.048735
	6	−1.431759	5.957982	−3.045955
	6	−2.708351	6.368690	−3.140314
	6	−3.213776	3.044662	−1.676796
	8	−3.124520	3.482980	−0.646699
C_{18}-CO_2	6	−5.008299	0.369070	−0.073057
	6	−3.861170	−0.032054	0.039363
	6	−2.520393	−0.064498	−0.049362
	6	−1.379446	0.279881	−0.312269
	6	−6.042219	1.165587	−0.394130
	6	−6.657625	2.124749	−0.830963
	6	−0.359305	1.026235	−0.769270

	6	−6.898666	3.312275	−1.412482
	6	0.242475	1.954267	−1.285131
	6	−6.693672	4.379241	−1.968443
	6	0.466667	3.130127	−1.896267
	6	0.248262	4.208570	−2.424254
	6	−6.031885	5.401470	−2.537295
	6	−5.104825	6.078271	−2.951972
	6	−0.430308	5.264398	−2.905123
	6	−3.849057	6.458986	−3.242675
	6	−1.367955	5.988643	−3.198079
	6	−2.631570	6.429507	−3.322622
	8	−3.148738	3.717308	−0.614183
	8	−3.304991	2.673999	−2.671089
	6	−3.226920	3.195774	−1.642697
C_{18}-SO_2	6	−5.035052	0.450242	0.013888
	6	−3.883933	0.056203	0.115442
	6	−2.542798	0.009513	0.034343
	6	−1.399656	0.352852	−0.220947
	6	−6.074394	1.246850	−0.292347
	6	−6.706125	2.203545	−0.712204
	6	−0.362635	1.085276	−0.660982
	6	−6.958975	3.394485	−1.283477
	6	0.243867	2.012912	−1.171250
	6	−6.757719	4.463376	−1.837896
	6	0.464692	3.187807	−1.783930
	6	0.237619	4.262442	−2.315302
	6	−6.088466	5.479211	−2.411298
	6	−5.150327	6.138346	−2.830788
	6	−0.463831	5.302602	−2.796659
	6	−3.890078	6.501743	−3.128004
	6	−1.405083	6.021734	−3.091307
	6	−2.672745	6.453845	−3.208535
	8	−3.082922	3.410485	−1.193903
	8	−2.531086	2.187805	−3.253133
	16	−3.499992	2.840530	−2.433712
C_{18}-SO_3	6	−4.978282	0.257410	0.044026
	6	−3.707638	−0.168251	0.147225
	6	−2.490227	−0.155505	0.065495
	6	−1.255273	0.300206	−0.204376
	6	−5.931793	0.974164	−0.214764
	6	−6.638227	2.030972	−0.652838
	6	−0.371180	1.052570	−0.580694
	6	−6.905072	3.115473	−1.146104
	6	0.217451	2.146985	−1.093208
	6	−6.753423	4.314936	−1.734466
	6	0.360044	3.249014	−1.599002
	6	0.066620	4.443737	−2.142075
	6	−6.213584	5.284514	−2.242754
	6	−5.246622	6.097082	−2.702064
	6	−0.578388	5.389295	−2.566906
	6	−4.125561	6.502836	−2.961993
	6	−1.625828	6.161974	−2.902793

	6	−2.783842	6.524136	−3.033437
	8	−3.274661	3.229509	−1.367976
	8	−3.379308	5.219131	0.062621
	8	−2.840981	3.038716	1.038603
	16	−3.164694	3.828639	−0.090315

c) Internal coordinates of ωB97XD/Def2TZVPP optimized complexes with single ring (ligand from the outer side)).

C_{18}-H_2	C	−3.86941500	0.03220400	0.04750600
	C	−2.54471800	−0.16094300	−0.07720700
	C	−1.37745600	0.04842800	−0.36576500
	C	−0.28543900	0.67456300	−0.83811800
	C	−4.96214500	0.57059100	−0.02792200
	C	−5.89973400	1.49234800	−0.30886100
	C	0.40986700	1.53391000	−1.35547500
	C	−6.40680400	2.52639100	−0.71292300
	C	0.75765400	2.68636900	−1.95443200
	C	−6.51881700	3.74564000	−1.26837600
	C	0.65473600	3.79319300	−2.45831400
	C	0.09688600	4.93228600	−2.90397400
	C	−6.20293000	4.79154700	−1.81230500
	C	−5.43686200	5.73800900	−2.38240200
	C	−0.75361100	5.76953300	−3.15914800
	C	−4.44582600	6.30663400	−2.81151200
	C	−1.95745300	6.36246900	−3.24280800
	C	−3.15995800	6.53748000	−3.12925500
	H	3.54269300	5.19875900	−2.83212400
	H	3.43700300	5.87129000	−3.13062000
C_{18}-CO	C	−4.94003900	0.17607800	−0.18116300
	C	−3.78242800	−0.20016200	−0.09030200
	C	−2.44148500	−0.19823100	−0.18402600
	C	−1.30799400	0.17878100	−0.43463300
	C	−5.99261600	0.96215700	−0.46658400
	C	−6.63671800	1.92154700	−0.85961300
	C	−0.30516400	0.96545700	−0.86307500
	C	−6.90731100	3.12706300	−1.38989000
	C	0.27217100	1.92866800	−1.34120800
	C	−6.73373600	4.22141600	−1.90167700
	C	0.46377300	3.13458300	−1.90378900
	C	0.21383300	4.22841100	−2.38361200
	C	−6.09640800	5.28166300	−2.42838200
	C	−5.18729900	5.99717500	−2.81717100
	C	−0.49400000	5.28628000	−2.81644400
	C	−3.94044000	6.41714800	−3.09426000
	C	−1.44834100	6.00114400	−3.07683600
	C	−2.72282800	6.41745900	−3.17796400
	C	3.86817200	1.56820900	−1.43863000
	O	4.69950300	0.86047000	−1.17639000
C_{18}-CO_2	C	−5.11209000	0.37738400	−0.06768100
	C	−3.98708400	−0.07382400	0.07441700
	C	−2.64689900	−0.16662700	0.01763500
	C	−1.48588000	0.12475000	−0.21994300
	C	−6.10203600	1.21766000	−0.41669700
	C	−6.66501200	2.20050200	−0.87115600

	C	−0.42308200	0.82377300	−0.65569300
	C	−6.84185200	3.39492500	−1.46269600
	C	0.23089900	1.72181000	−1.16036400
	C	−6.57989800	4.44980600	−2.01772000
	C	0.51728100	2.88514600	−1.77121500
	C	0.35670000	3.96962000	−2.30824500
	C	−5.86057800	5.43982500	−2.57476500
	C	−4.89571700	6.07386800	−2.97030200
	C	−0.26138800	5.05355700	−2.80975600
	C	−3.61672200	6.39588700	−3.23188000
	C	−1.16087200	5.81480900	−3.12644800
	C	−2.40029800	6.31202900	−3.28307600
	O	3.94218400	2.67835100	−1.96681900
	O	3.64031200	4.72638600	−2.99567700
	C	3.78856900	3.70214900	−2.48088000
C_{18}-SO_2	C	−4.65459200	0.54600100	0.41692800
	C	−3.49047800	0.18019300	0.39886800
	C	−2.17318900	0.17958200	0.12969200
	C	−1.08915500	0.53394900	−0.30392800
	C	−5.74920900	1.29378700	0.19256000
	C	−6.44849700	2.20809900	−0.21282300
	C	−0.16895600	1.28064300	−0.93943500
	C	−6.80696800	3.35073500	−0.82405200
	C	0.32602100	2.19062500	−1.58610200
	C	−6.71585200	4.38639600	−1.46315100
	C	0.43100300	3.33064400	−2.29155100
	C	0.10389500	4.36715400	−2.84591900
	C	−6.17112000	5.38858000	−2.17487900
	C	−5.33225000	6.06287100	−2.75022300
	C	−0.66550400	5.37545700	−3.29220000
	C	−4.13908700	6.45568500	−3.22951700
	C	−1.66019800	6.05277900	−3.49487300
	C	−2.94318400	6.45418500	−3.47276400
	O	2.60720300	−1.05459500	−0.91229400
	O	3.56827400	0.73489600	−2.29539700
	S	3.18976000	0.24432200	−1.01005000
C_{18}-SO_3	C	−4.98726300	0.28394300	−0.00370100
	C	−3.71159100	−0.12958900	0.09417300
	C	−2.49413100	−0.12965300	0.01163700
	C	−1.24983500	0.29896400	−0.26398100
	C	−5.94300900	0.99885300	−0.25764100
	C	−6.65083300	2.06003000	−0.68282600
	C	−0.33682800	1.01340100	−0.64452100
	C	−6.90024800	3.15731500	−1.15482800
	C	0.29028500	2.08345700	−1.16398600
	C	−6.70777700	4.36794600	−1.70733900
	C	0.47500300	3.17988900	−1.66541100
	C	0.18974600	4.38764200	−2.18427400
	C	−6.13458200	5.33702800	−2.17777400
	C	−5.13175000	6.12794600	−2.59831500
	C	−0.45075800	5.35354100	−2.57143300
	C	−4.00277200	6.51731800	−2.84834800

	C	−1.50248100	6.14432500	−2.85203800
	C	−2.66104700	6.51530600	−2.93883500
	O	3.25256000	5.30886100	−2.80570100
	O	2.00501100	6.52029700	−4.53361100
	O	2.17050300	7.44182500	−2.26777500
	S	2.46163200	6.41556200	−3.19801000
d) Internal coordinates of ωB97XD/Def2TZVPP optimized covalent complexes with single ring (ligand within).				
C_{18}-H_2	6	−4.191998	0.329990	−0.176284
	6	−2.855539	0.419579	−0.351684
	6	−1.729353	0.781656	−0.628206
	6	−0.566071	1.363121	−1.005947
	6	−5.384138	0.573056	−0.163912
	6	−6.517911	1.280261	−0.364254
	6	0.382315	2.012317	−1.388647
	6	−7.179707	2.237171	−0.723919
	6	1.444825	2.809190	−1.845369
	6	−7.331738	3.469549	−1.258318
	6	1.293276	4.041141	−2.381129
	6	0.053965	4.680415	−2.549095
	6	−6.912525	4.497303	−1.759352
	6	−5.969953	5.342203	−2.231896
	6	−1.036935	5.193049	−2.669636
	6	−4.855854	5.736931	−2.520827
	6	−2.323001	5.613707	−2.724914
	6	−3.519147	5.824067	−2.695347
	1	2.445328	2.403285	−1.757731
	1	2.177756	4.576929	−2.703554
C_{18}-CO	6	−4.322154	−0.508354	−0.214409
	6	−2.974016	−0.434462	−0.217232
	6	−1.864763	0.045960	−0.355625
	6	−0.867833	0.916839	−0.621978
	6	−5.470155	−0.128227	−0.354962
	6	−6.456411	0.744485	−0.653448
	6	−0.246985	1.913484	−0.935080
	6	−6.935845	1.807233	−1.004048
	6	0.157705	3.143045	−1.327373
	6	−6.955087	3.090999	−1.421756
	6	0.274045	4.291077	−1.698376
	6	0.151162	5.601486	−2.125601
	6	−6.529197	4.176272	−1.769916
	6	−5.687332	5.184621	−2.082459
	6	−0.846083	6.484377	−2.428559
	6	−4.686039	5.844223	−2.280404
	6	−2.229332	6.507355	−2.458483
	6	−3.425350	6.315696	−2.413885
	6	0.514482	6.923158	−2.548993
	8	1.327321	7.753197	−2.806423
C_{18}-CO_2	6	−4.411051	−0.303574	−0.189034
	6	−3.200096	−0.180071	−0.209889
	6	−1.981336	0.369370	−0.384495
	6	−1.039941	1.097700	−0.633257
	6	−5.722468	−0.017872	−0.321106

	6	−6.674265	0.689547	−0.600746
	6	−0.221718	2.111498	−0.986156
	6	−7.324096	1.794882	−1.019413
	6	0.372128	3.107678	−1.341241
	6	−7.372008	2.937763	−1.439538
	6	0.768920	4.356068	−1.790835
	6	0.117234	5.478595	−2.213372
	6	−6.921366	4.133777	−1.869433
	6	−6.102745	4.985702	−2.164543
	6	−1.210051	5.835438	−2.371078
	6	−4.940811	5.635661	−2.378698
	6	−2.411972	5.978781	−2.449934
	6	−3.761923	5.923852	−2.458162
	8	2.057998	7.012886	−2.731051
	6	1.485291	6.052405	−2.394388
	8	2.068706	4.810834	−1.930879
C_{18}-SO_2	6	−4.553354	−0.654066	−0.253337
	6	−3.205727	−0.589419	−0.231151
	6	−2.102287	−0.087885	−0.344926
	6	−1.107490	0.790300	−0.589252
	6	−5.696249	−0.264808	−0.415117
	6	−6.622772	0.663355	−0.734836
	6	−0.472543	1.786767	−0.876330
	6	−7.038857	1.752100	−1.088142
	6	−0.017328	3.004571	−1.245107
	6	−6.923421	3.037054	−1.485045
	6	0.194938	4.149601	−1.586594
	6	0.287753	5.458776	−2.013019
	6	−6.416598	4.098286	−1.797266
	6	−5.487968	5.041858	−2.060216
	6	−0.615455	6.418188	−2.353570
	6	−4.464273	5.675418	−2.223729
	6	−2.013022	6.391890	−2.366540
	6	−3.194673	6.130567	−2.323353
	8	2.517239	6.563798	−3.122811
	8	0.124678	7.522436	−2.627049
	16	1.571621	6.728409	−2.050837
C_{18}-SO_3	6	−4.682579	−0.758748	−0.323598
	6	−3.335402	−0.693833	−0.298515
	6	−2.227042	−0.202557	−0.407864
	6	−1.231622	0.679305	−0.634186
	6	−5.823072	−0.359496	−0.477757
	6	−6.749883	0.572960	−0.781613
	6	−0.584809	1.672224	−0.905571
	6	−7.163832	1.668250	−1.117071
	6	−0.128667	2.896486	−1.250230
	6	−7.048349	2.959202	−1.491924
	6	0.095890	4.042594	−1.576907
	6	0.165758	5.364992	−1.962070
	6	−6.541554	4.024798	−1.789572
	6	−5.610442	4.969464	−2.035937
	6	−0.739826	6.330106	−2.274024

	6	−4.585410	5.604053	−2.188403
	6	−2.129824	6.306183	−2.311877
	6	−3.313253	6.051029	−2.277978
	8	2.135146	6.402922	−3.491502
	8	2.060604	7.097539	−1.122971
	16	1.432622	6.574660	−2.276666
	8	0.011197	7.439288	−2.575223

e) Internal coordinates of ωB97XD/Def2TZVPP optimized complexes with double ring.

$(C_{18})_2$-H_2	6	−5.374816	−0.099284	−0.334556
	6	−4.239354	−0.516068	−0.171113
	6	−2.895721	−0.551221	−0.166142
	6	−1.739288	−0.192400	−0.319261
	6	−6.380898	0.730649	−0.658853
	6	−6.962445	1.729882	−1.050425
	6	−0.690927	0.585960	−0.637857
	6	−7.163331	2.970700	−1.525858
	6	−0.057944	1.552610	−1.031489
	6	−6.923239	4.092243	−1.943169
	6	0.206055	2.777985	−1.516143
	6	0.020541	3.905395	−1.945434
	6	−6.227643	5.173776	−2.333298
	6	−5.276808	5.894122	−2.592322
	6	−0.622013	5.015854	−2.345325
	6	−4.003842	6.296135	−2.746256
	6	−1.535385	5.783338	−2.603183
	6	−2.783880	6.258208	−2.749371
	6	−4.408815	2.207309	2.353352
	6	−3.065632	2.169312	2.346729
	6	−1.908205	2.532754	2.211699
	6	−0.857169	3.321544	1.930745
	6	−5.544082	2.637034	2.226218
	6	−6.549120	3.482607	1.943335
	6	−0.217414	4.300013	1.579711
	6	−7.131646	4.491036	1.578262
	6	0.053283	5.534123	1.121953
	6	−7.326297	5.731943	1.100662
	6	−0.124427	6.661397	0.688962
	6	−0.765094	7.760460	0.256098
	6	−7.081096	6.842130	0.656281
	6	−6.378078	7.903186	0.225304
	6	−1.679291	8.511097	−0.045001
	6	−5.422829	8.604283	−0.067314
	6	−2.929377	8.970440	−0.224376
	6	−4.149339	9.000424	−0.232498
	1	−4.327497	5.186426	0.247400
	1	−3.637120	5.001977	0.455105
$(C_{18})_2$-CO	6	−5.226929	0.136703	−0.244967
	6	−4.102857	−0.317503	−0.104582
	6	−2.762095	−0.401414	−0.136592
	6	−1.600098	−0.081504	−0.328136
	6	−6.213774	0.993629	−0.556943
	6	−6.781468	1.999431	−0.951564

	6	−0.543176	0.662079	−0.696671
	6	−6.971066	3.232564	−1.450781
	6	0.097066	1.604906	−1.133180
	6	−6.731961	4.333917	−1.919661
	6	0.364300	2.811557	−1.660531
	6	0.189203	3.925209	−2.128210
	6	−6.030885	5.379728	−2.390230
	6	−5.071678	6.056740	−2.724921
	6	−0.440479	5.030485	−2.561172
	6	−3.791177	6.403778	−2.941435
	6	−1.337005	5.810827	−2.838755
	6	−2.573897	6.318161	−2.975783
	6	−4.617920	2.181261	2.602947
	6	−3.277173	2.100014	2.554018
	6	−2.116577	2.420465	2.351926
	6	−1.062600	3.169882	1.985052
	6	−5.744206	2.636285	2.482395
	6	−6.741117	3.486585	2.183844
	6	−0.429713	4.124364	1.562230
	6	−7.317416	4.487348	1.788740
	6	−0.164095	5.339108	1.052210
	6	−7.517393	5.712819	1.275086
	6	−0.345601	6.456880	0.596740
	6	−0.977608	7.560952	0.163937
	6	−7.276334	6.807057	0.791273
	6	−6.576857	7.849345	0.311686
	6	−1.882734	8.328502	−0.121345
	6	−5.619760	8.527190	−0.025520
	6	−3.124121	8.820550	−0.270812
	6	−4.342530	8.884269	−0.241960
	6	−3.580962	4.269907	−0.166614
	8	−3.838097	5.131806	0.506206
$(C_{18})_2$-CO_2	6	−4.652229	−0.223726	−0.139669
	6	−3.458146	−0.468320	−0.079285
	6	−2.129379	−0.309254	−0.198413
	6	−1.058156	0.213477	−0.459662
	6	−5.791164	0.447890	−0.377454
	6	−6.548858	1.340412	−0.722319
	6	−0.174860	1.134640	−0.879564
	6	−6.971564	2.526473	−1.191096
	6	0.266353	2.176090	−1.337982
	6	−6.949985	3.658648	−1.646568
	6	0.291330	3.415100	−1.856760
	6	−0.095502	4.489968	−2.286394
	6	−6.464586	4.817441	−2.123624
	6	−5.665047	5.663377	−2.491882
	6	−0.931690	5.477534	−2.648193
	6	−4.485149	6.241451	−2.775934
	6	−1.964170	6.097989	−2.845855
	6	−3.277559	6.379589	−2.890628
	6	−5.119909	2.378358	2.488057
	6	−3.929298	2.112330	2.533351

	6	−2.599355	2.263536	2.409857
	6	−1.526107	2.786423	2.154491
	6	−6.257632	3.060881	2.274653
	6	−7.006519	3.965192	1.941355
	6	−0.638174	3.711484	1.751356
	6	−7.432839	5.148596	1.469216
	6	−0.200124	4.765275	1.317974
	6	−7.400414	6.272257	0.993951
	6	−0.161145	6.012861	0.820333
	6	−0.546155	7.090629	0.396374
	6	−6.914300	7.420695	0.494278
	6	−6.106377	8.251890	0.112805
	6	−1.370519	8.081332	0.017077
	6	−4.924772	8.825388	−0.170178
	6	−2.404916	8.690344	−0.203530
	6	−3.717338	8.969250	−0.272325
	8	−3.372617	3.357803	−0.859602
	8	−3.777210	5.167048	0.521759
	6	−3.574989	4.262535	−0.169092
$(C_{18})_2$-SO_2	6	−5.243707	0.443939	−0.178258
	6	−4.004233	−0.046688	−0.009601
	6	−2.786254	−0.114687	−0.045227
	6	−1.504237	0.210413	−0.280916
	6	−6.158117	1.187015	−0.497440
	6	−6.792061	2.264554	−0.991210
	6	−0.540021	0.857601	−0.655705
	6	−6.975348	3.352414	−1.514356
	6	0.179053	1.861105	−1.185039
	6	−6.690393	4.533137	−2.091490
	6	0.433013	2.928225	−1.719744
	6	0.241297	4.130365	−2.287085
	6	−6.033425	5.451017	−2.558676
	6	−4.969144	6.172396	−2.953815
	6	−0.331473	5.115107	−2.724479
	6	−3.801366	6.468568	−3.153775
	6	−1.335648	5.944109	−3.053853
	6	−2.460136	6.395938	−3.198870
	6	−4.380697	2.527151	2.658477
	6	−3.162221	2.459658	2.600120
	6	−1.885030	2.795059	2.345042
	6	−0.935005	3.456339	1.956083
	6	−5.626093	3.013658	2.513741
	6	−6.521743	3.779170	2.193337
	6	−0.215185	4.460228	1.427087
	6	−7.143491	4.862682	1.697059
	6	0.033721	5.531980	0.898964
	6	−7.294661	5.950890	1.164835
	6	−0.135179	6.738818	0.334283
	6	−0.700797	7.726225	−0.106476
	6	−7.019140	7.125238	0.572775
	6	−6.368623	8.035940	0.085997
	6	−1.691639	8.565118	−0.450286

	6	−5.319431	8.764481	−0.330291
	6	−2.819713	9.006366	−0.598397
	6	−4.160321	9.076042	−0.550458
	8	−2.915233	3.596719	−1.062584
	8	−3.352922	5.579990	0.317894
	16	−3.794834	4.329414	−0.209781
$(C_{18})_2$-SO_3	6	−5.997191	0.690783	−0.805054
	6	−4.938868	0.206346	−0.439041
	6	−3.613412	0.030851	−0.291389
	6	−2.408161	0.191965	−0.394744
	6	−6.846123	1.535571	−1.417421
	6	−7.210261	2.487502	−2.088018
	6	−1.213676	0.694653	−0.755706
	6	−7.111488	3.592044	−2.849287
	6	−0.366102	1.402884	−1.274504
	6	−6.603491	4.507993	−3.475228
	6	0.196573	2.387074	−1.998452
	6	0.284130	3.371122	−2.714421
	6	−5.667620	5.305463	−4.020908
	6	−4.584510	5.765624	−4.344248
	6	−0.117327	4.416532	−3.459640
	6	−3.256162	5.932640	−4.475918
	6	−0.886723	5.194784	−3.998923
	6	−2.054310	5.751216	−4.368337
	6	−3.714439	2.101358	2.659982
	6	−2.385825	2.264842	2.525381
	6	−1.301885	2.724387	2.202971
	6	−0.365203	3.522523	1.659489
	6	−4.915573	2.288434	2.553191
	6	−6.082736	2.848262	2.188402
	6	0.138093	4.442469	1.035545
	6	−6.850340	3.628581	1.649827
	6	0.235247	5.547878	0.275849
	6	−7.251679	4.673416	0.904405
	6	−0.130529	6.499246	−0.394414
	6	−0.978963	7.341495	−1.010172
	6	−7.168502	5.655505	0.185365
	6	−6.605892	6.636168	−0.543193
	6	−2.034493	7.828240	−1.381019
	6	−5.760194	7.342611	−1.067820
	6	−3.359535	8.002764	−1.533134
	6	−4.565329	7.843390	−1.430813
	8	−2.369611	4.327475	−1.280031
	8	−3.434404	5.223566	0.740645
	16	−3.473858	4.385127	−0.398456
	8	−4.618219	3.597944	−0.663907
